# Right ventricular myocardial deoxygenation in patients with pulmonary artery hypertension

**DOI:** 10.1186/s12968-020-00694-0

**Published:** 2021-03-08

**Authors:** Karthigesh Sree Raman, Ranjit Shah, Michael Stokes, Angela Walls, Richard J. Woodman, Rebecca Perry, Jennifer G. Walker, Susanna Proudman, Carmine G. De Pasquale, David S. Celermajer, Joseph B. Selvanayagam

**Affiliations:** 1 College of Medicine and Public Health, Flinders University, Adelaide, Australia; 2 Department of Cardiovascular Medicine, Flinders Medical Centre, Adelaide, South Australia 5042 Australia; 3 Cardiac Imaging Research, South Australian Health & Medical Research Institute, Adelaide, Australia; 4 Department of Medicine (Northland Campus), Faculty of Medicine and Health Sciences, University of Auckland, Auckland, New Zealand; 5 Department of Cardiology, Royal Adelaide Hospital, Adelaide, Australia; 6 Clinical Research and Imaging Centre, South Australian Health & Medical Research Institute, Auckland, Australia; 7 Flinders Centre of Epidemiology and Biostatistics, College of Medicine and Public Health, Flinders University, Adelaide, Australia; 8 Rheumatology Unit, Royal Adelaide Hospital and Discipline of Medicine, University of Adelaide, Adelaide, Australia; 9 Sydney Medical School, University of Sydney and Royal Prince Alfred Hospital, Sydney, Australia; 10 Department of Cardiology, Royal Prince Alfred Hospital, Camperdown, Australia

**Keywords:** Pulmonary artery hypertension, Right ventricle, Cardiac magnetic resonance (CMR), Coronary microvascular dysfunction, Oxygen-sensitive cardiac magnetic resonance, T1 mapping

## Abstract

**Background:**

In pulmonary arterial hypertension (PAH), progressive right ventricular (RV) dysfunction is believed to be largely secondary to RV ischaemia. A recent pilot study has demonstrated the feasibility of Oxygen-sensitive (OS) cardiovascular magnetic resonance (CMR) to detect in-vivo RV myocardial oxygenation. The aims of the present study therefore, were to assess the prevalence of RV myocardial ischaemia and relationship with RV myocardial interstitial changes in PAH patients with non-obstructive coronaries, and corelate with functional and haemodynamic parameters.

**Methods:**

We prospectively recruited 42 patients with right heart catheter (RHC) proven PAH and 11 healthy age matched controls. The CMR examination involved standard functional imaging, OS-CMR imaging and native T1 mapping. An ΔOS-CMR signal intensity (SI) index (stress/rest signal intensity) was acquired at RV anterior, RV free-wall and RV inferior segments. T1 maps were acquired using Shortened Modified Look-Locker Inversion recovery (ShMOLLI) at the inferior RV segment.

**Results:**

The inferior RV ΔOS-CMR SI index was significantly lower in PAH patients compared with healthy controls (9.5 (– 7.4–42.8) vs 12.5 (9–24.6)%, *p* = 0.02). The inferior RV ΔOS-CMR SI had a significant correlation to RV inferior wall thickness (r = – 0.7, *p* < 0.001) and RHC mean pulmonary artery pressure (mPAP) (r = – 0.4, *p* = 0.02). Compared to healthy controls, patients with PAH had higher native T1 in the inferior RV wall: 1303 (1107–1612) vs 1232 (1159–1288)ms, *p* = 0.049. In addition, there was a significant difference in the inferior RV T1 values between the idiopathic PAH and systemic sclerosis associated PAH patients: 1242 (1107–1612) vs 1386 (1219–1552)ms, *p* = 0.007.

**Conclusion:**

Blunted OS-CMR SI suggests the presence of in-vivo microvascular RV dysfunction in PAH patients. The native T1 in the inferior RV segments is significantly increased in the PAH patients, particularly among the systemic sclerosis associated PAH group.

## Introduction

Pulmonary arterial hypertension (PAH) is a progressive disorder that affects both the pulmonary vasculature and the heart [1]. It is a rare but serious disease, affecting up to 50 per million population [2]. Although the initial insult in PAH involves the pulmonary vasculature, prognosis of patients with PAH is most dependent on right ventricular (RV) size and function [3]. While there are several putative causes, RV ischemia is thought to be a significant driver [4]. The increasing pulmonary artery pressure decreases the pressure gradient between the aorta and the RV, thereby reducing myocardial blood flow in the right coronary artery which, leads to myocardial ischemia [5, 6]. In addition, the RV adapts to the increased afterload by increasing wall thickness and contractility. However, RV hypertrophy without a corresponding increase in the cross-sectional area of the right coronary artery can exacerbate ischemia of the RV [7]. RV adaptive changes due to the elevated pulmonary artery pressures affects the integrity of the collagen network of the cardiomyocyte and interstitium leading to RV fibrosis [8]. Whether the adaptive changes in the myocardial interstitium has any causal relationship to myocardial ischaemia in PAH patients remains unknown.

Oxygen-sensitive (OS) cardiovascular magnetic resonance (OS-CMR), also known as blood oxygen level dependent (BOLD) CMR enables the in-vivo assessment myocardial oxygenation at the tissue level [9–13]. OS-CMR utilises the natural paramagnetic properties of haemoglobin [14]. Following vasodilator stress, the alterations to the deoxygenated haemoglobin concentration act as an endogenous contrast agent leading to signal intensity (SI) changes in OS-CMR sequence. CMR native T1 mapping can be used to characterise changes in the interstitial myocardium of the left ventricle (LV) [15]. T1 mapping quantifies the T1 relaxation time of each voxel of an image being quantified. Different tissues each have distinctive T1 relaxation times and this can vary with a change in tissue composition. CMR T1 mapping has been used for inflammation/oedema imaging and serves as a surrogate for diffuse interstitial fibrosis in the absence of an alternative cause of interstitial expansion (oedema, infiltrations/fibre disarray) [16]. Previous studies have demonstrated higher T1 values in the RV of patients with PAH [17], however to our knowledge no study has looked at the relationship between interstitial myocardial changes in PAH and myocardial ischaemia. Furthermore, in a recent small proof of concept study, we have demonstrated the feasibility of OS-CMR technique in detecting myocardial oxygenation abnormalities in the RV of 20 patients with PAH [18]. However, the sample size was too small to look at the relationship between myocardial oxygenation and functional or haemodynamic indices. Hence the aim of the present study was to assess the prevalence of RV myocardial ischaemia and its relationship with changes in the myocardial interstitium of patients with known PAH and with non-obstructive coronaries using OS-CMR and native T1 mapping.

## Methods

### Study population

Patients undergoing treatment in the PAH clinics at two South Australian hospitals were invited to participate in this study. Inclusion criteria included right heart catheter (RHC) proven PAH (defined as pulmonary capillary wedge pressure (PCWP) $\le$ 15 mmHg, and mean pulmonary artery pressure (mPAP) ≥ 25 mmHg). Exclusion criteria included severe RV dysfunction on echocardiography (determined by tricuspid annular plane systolic excursion < 1.7), echocardiographic LV ejection fraction < 50% and/or coronary artery disease (defined as > 70% luminal stenosis in an epicardial coronary artery at angiography or prior myocardial infarction) as well as contraindications to CMR and/or adenosine (second or third degree heart block, obstructive pulmonary disease or dipyridamole use). Eleven healthy subjects also underwent the same CMR imaging procedures and served as a control group. The healthy subjects had no known cardiac/respiratory disease or symptoms and were free of any cardiac risk factors, including hypertension, smoking and diabetes. This study was approved by the Southern Adelaide Clinical Human Research Ethics Committee (HREC/15/SAC/397), and all participants provided written and informed consent to participate in the study.

### CMR protocol

**Fig. 1:**
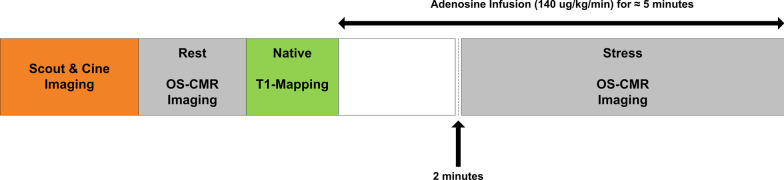
CMR Imaging Protocol. Cine, Oxygen sensitive cardiovascular magnetic resonance (OS-CMR) and native T1 mapping imaging protocol for pulmonary artery hypertension (PAH) and healthy control groups

The full protocol has been described elsewhere [18]; in brief, CMR was performed using a 3 T clinical CMR scanner (MAGNETOM Skyra, Siemens Healthineers, Erlangen, Germany) with 18 channel torso phased array coil in conjunction with a spinal coil posteriorly). Electrographically (ECG) gated balanced steady-state free precession (bSSFP) sequence was used to acquire cine images in vertical and horizontal long-axis, and ten short-axis images covering the entire RV and LV. For OS imaging, a single mid-ventricular slice was acquired at mid-diastole using a single-shot T2-prepared ECG-gated bSSFP sequence (FOV of 340 × 340 cm, matrix 168 × 192 m, slice thickness 6 mm, producing a voxel size 2.02 × 1.77 mm. The repetition time (TR) was 256 ms and with echo time (TE) of 1.21 ms, flip angle 44˚). Each OS-CMR image was acquired during a single breath-hold over six heart beats. Prior to adenosine infusion, four resting OS-CMR images were acquired. Stress OS-CMR stress images acquisition commenced 2 min after adenosine infusion. Four to six OS-CMR images were acquired over the stress period with the typical duration of stress OS-CMR image acquisition was 3 min with the total adenosine infusion duration of about 5 min (Fig. 1).

Native T1 mapping were acquired using Shortened Modified Look-Locker Inversion recovery (ShMOLLI) on a single mid-ventricular short-axis slice as previously described [19]. The ShMOLLI sequence was performed with prospective ECG triggering performed until optimal image quality was obtained. This sequence has a FOV of 360 × 360 cm, matrix 192 × 144, slice thickness 8 mm, producing an interpolated voxel size 0.9 × 0.9 mm. The TR was 379.40 and with TE of 1.07. Typically, the flip angle was set to 35 degrees and IPAT (GRAPPA) factor of 2, with inversion time (TI) of 260 ms. The T1 maps were acquired after the resting OS-CMR images prior to adenosine infusion (Fig. 1). The mid-ventricular slice location selected was matched to the selected mid-ventricular resting OS-CMR images. Stress heart rate and blood pressure were obtained every minute of adenosine infusion. Patients were monitored throughout the study by ECG, sphygmomanometry and pulse oximetry.

### CMR analysis

CMR analysis was performed using cvi^42^ (Circle CVI, Calgary, Canada). The short-axis bSSFP cine images were used to quantify LV and RV systolic function. Standard function parameters were calculated including LV ejection fraction (LVEF), RV ejection fraction (RVEF), cardiac index, and body surface area (BSA) indexed end-diastolic (EDVI), end-systolic (ESVI) volumes for LV/RV and LV indexed myocardial mass. End-diastolic RV mass and volumes was used to calculate RV mass/volume ratio.

The OS-CMR assessment of the RV and LV was performed using cvi^42^ (Circle Cardiovascular Imaging, Calgary, Canada) as previously described [18]. In brief the RV was segmented into RV anterior, RV free-wall and RV inferior segments. OS-CMR analysis of RV anterior and RV free-wall were limited by thinned myocardium and partial volume effects [18]. Reliable OS signal was only obtained in RV inferior [18], hence a region of interest (ROI) contour was traced manually in this RV segment. For the LV OS-CMR assessment, the epicardium and endocardium borders were manually traced and the LV was sub-divided into 6 equiangular segments based on a standard American Heart Association (AHA) segmentation of the mid-ventricular slice as previously reported [20]. The mean myocardial Signal Intensity (SI) within the RV and LV segments was obtained as previously described [18]. As it is a cardiac gated sequence, the mean signal intensity at rest and stress was corrected for the variations of heart rate and its effects on T1 relaxation as previously described [21]. The relative SI change was calculated as ΔSI (%) = (SI stress-SI rest)/SI rest × 100. Reproducibility of the OS-CMR of the RV has been previously published [18].

**Fig. 2:**
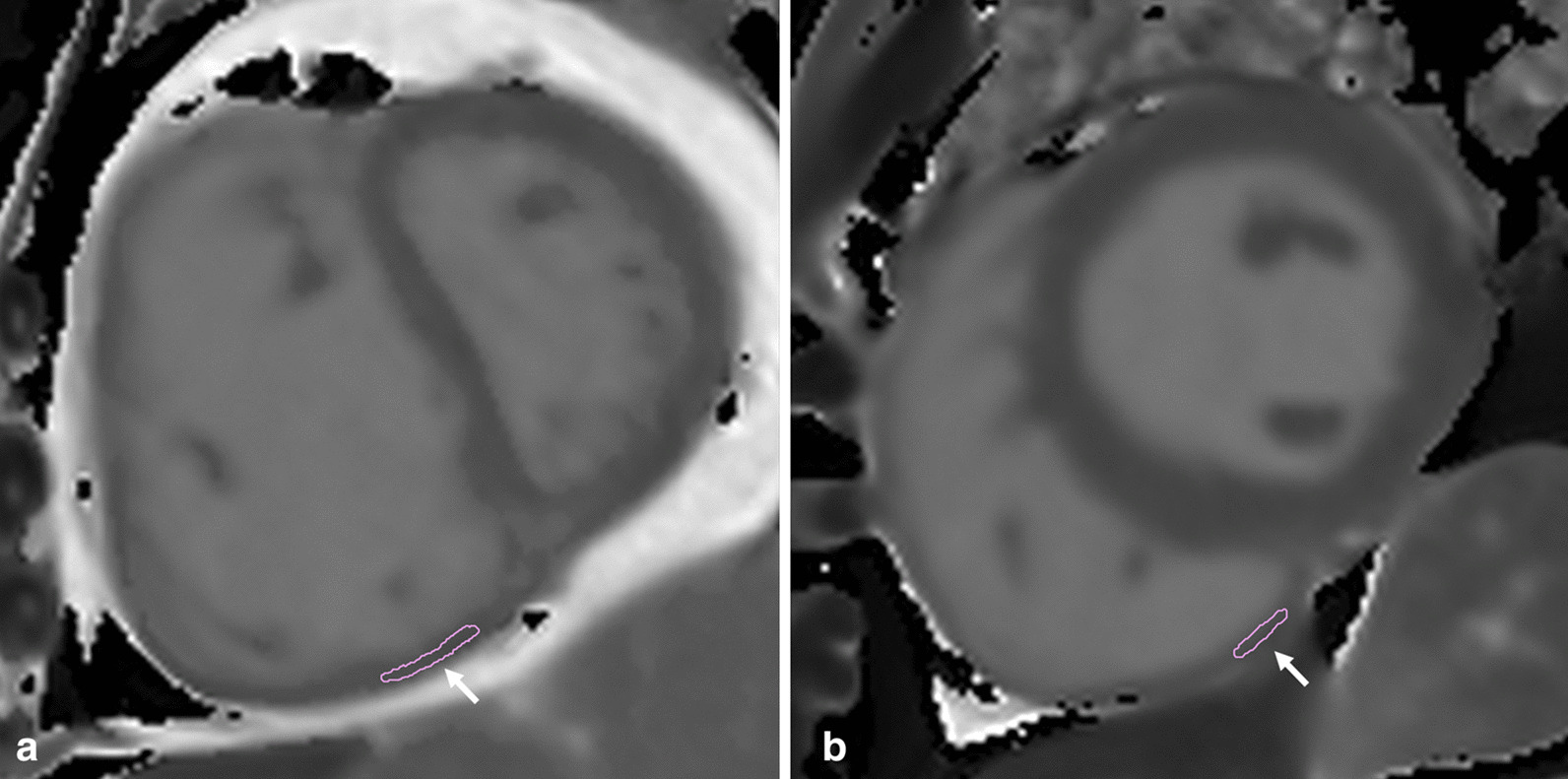
Representative case inferior wall right ventricular (RV) native T1-mapping in Pulmonary Artery Hypertension (PAH) and healthy control group. Representative case with the region of interest (ROI) contour (white arrow) traced manually on the T1 maps of inferior segment of RV in **a** PAH and **b** healthy control groups

The T1-values of the RV were assessed using cvi^42^ (Circle Cardiovascular Imaging). As previously described [22], a ROI contour was traced manually on the T1 maps of inferior RV region avoiding the RV insertion point (Fig. 2); attempts were made to draw ROI in RV anterior and RV free-wall regions but this was not feasible. The ROI was carefully drawn within myocardial walls to avoid partial volumes and epicardial fat (Fig. 2). For the LV, the endocardial and epicardial contours were manually traced and was sub-divided into 6 equiangular segments based on a standard AHA segmentation [20]. A mean myocardial T1 value was obtained within each segment.

### Statistical analysis

All analyses were performed using the Stata statistical software version 15.1 (StataCorporation, College Station, Texas, USA). Categorical data are described using frequencies and percentages and continuous data using median and range. Differences between groups in the mean change in myocardial oxygenation response and native T1-values were assessed using Mann–Whitney test. Changes in these same outcomes within patient groups were assessed using the Sign-rank test. Reproducibility of measurements for inter and intra-observers was assessed by using the coefficient of variation and by assessing the absolute and proportional bias. Absolute bias was assessed using a t-test of the differences in the 2 measures versus zero and proportional bias by regressing the differences in the 2 measures on the mean for the 2 measurements. Associations between test parameters were assessed using the Spearman’s rho (ρ) correlation coefficient. A 2-sided Type 1 error rate of alpha = 0.05 was used for assessing statistical significance.

## Results

### Participants characteristics

The subject characteristics are summarised in Table 1. Both PAH and healthy control groups were well matched for age and gender. The aetiology within PAH were idiopathic pulmonary artery hypertension (IPAH) (n = 20, 48%), systemic sclerosis associated PAH (SScPAH) (n = 17, 40%) and chronic thromboembolic pulmonary hypertension (CTEPH) (n = 5, 12%). All patients had RHC and coronary angiogram proven diagnosis of PAH with a median (range) mPAP of 33 (16–67) mmHg and PCWP of 12 (4–15) mmHg. Mean duration between RHC with coronary angiogram and CMR study was 17 months. The mean 6 min walk distance in the patient group was 384 (100–600) meters. World Health Organisation functional class was between class II (n = 27, 64%) and III (n = 15, 36%). The PAH patients had an echocardiography-estimated resting systolic pulmonary artery pressure of 41 (27–128) mmHg. Furthermore, on echocardiography 8 patients (19%) had mild RV dilatation and 5 patients (12%) had moderate RV dilation. All PAH patients were receiving treatment with pulmonary vasodilators (e.g., bosentan, macitentan, sildenafil, riociguat) or a combination of macitentan and sildenafil. At recruitment, the PAH patients were stable and the pulmonary vasodilators therapy was continued prior to the CMR research scan.

**Table 1:** Patient demographics and baseline clinical data

Variables	PAH (n = 42)	Healthy Controls (n = 11)	*p-*value
Age (years), median (range)	71 (63–79)	66 (60–69)	0.123
Females sex, n (%)	27 (68%)	4 (36%)	0.061
Comorbidities			
Hypertension, n (%)	22 (52%)	0	
Diabetes, n (%)	7 (17%)	0	
Dyslipidaemia, n(%)	13 (31%)	0	
Chronic Obstructive Airways Disease, n (%)	10 (24%)	0	
Obstructive Sleep Apnoea, n (%)	11 (26%)	0	
Atrial Fibrillation, n (%)	8 (19%)	0	
Right heart catheter haemodynamic indices			
Mean pulmonary artery pressures (mmHg), median (range)	33 (16–57)	0	
Pulmonary artery wedge pressure (mmHg), median (range)	12 (4–15)	0	
Mean right atrial pressures (mmHg), median (range)	12 (2–18)	0	
Cardiac index (L/min/m^2^), median (range)	3 (2–4)	0	
Pulmonary vascular resistance index (Woods unit m^2^), median (range)	7 (3–28)	0	
Medication			
Aspirin, n (%)	6 (14%)	0	
Beta-blockers, n (%)	10 (24%)	0	
ACEi/ARB, n (%)	12 (29%)	0	
Statins, n (%)	8 (19%)	0	
Calcium channel blockers	13 (31%)	0	
Endothelin receptor blockers, n (%)	34 (81%)	0	
PDE5 inhibitor, n (%)	18 (43%)	0	
Soluble guanylate cyclase, n (%)	3 (7%)	0	
Combination therapy, n (%)^a^	14 (33%)	0	

^a^Combination therapy of a dual therapy compromising of either endothelin receptor blocker, PDE5 inhibitor or soluble guanylate cyclase

*ACEi* angiotensin-converting enzyme inhibitors, *ARB* angiotensin II receptor blockers, *PDE5* phosphodiesterase type 5 inhibitor

### CMR characteristics

Table 2 shows a comparison of CMR variables between PAH patients and controls. The only significant difference in CMR volumetric and functional indices between PAH and healthy controls were the RV mass index (17 (9–42) vs 13 (9–19)g/m^2^, p = 0.001), RV mass/volume ratio (0.30 (0.22–0.49) vs 0.22 (0.15–0.25), p < 0.001) and inferior RV wall thickness (4 (3–9) vs 3 (2–5)mm, p = 0.002). Table 3 demonstrates the CMR variables between the IPAH and SScPAH subgroups. The inferior RV wall thickness was measured on the short axis cine slice location similar to the mid ventricular slice location chosen for the OS imaging.

**Table 2:** CMR ventricular function in pulmonary hypertension and healthy control groups

Variables	PAH (n = 42)	Healthy Controls (n = 11)	*p-*value
LVEF (%), median (range)	70 (46–86)	69 (51–75)	0.875
LV EDVI (ml/m^2^), median (range)	70 (44–112)	65 (45–81)	0.302
LV ESVI (ml/m^2^), median (range)	21 (7–52)	20 (13–28)	0.645
LV mass index (g/m^2^), median (range)	51 (36–72)	48 (39–68)	0.280
LV SV index (ml/m^2^), median (range)	46 (32–71)	45 (29–58)	0.428
RVEF (%), median (range)	61 (45–79)	63 (50–79)	0.304
RV EDVI (ml/m^2^), median (range)	60 (30–111)	60 (47–89)	0.972
RV ESVI (ml/m^2^), median (range)	22 (12–61)	22 (10–41)	0.823
RV SV Index (ml/m^2^), median (range)	37 (15–60)	38 (27–54)	0.595
RV ED Mass Index (g/m^2^), median (range)	17 (9–42)	13 (9–19)	0.001
RV (ED) mass/volume ratio, median (range)	0.30 (0.22–0.49)	0.22 (0.15–0.25)	< 0.001
RV inferior wall thickness (mm), median (range)	4 (3–9)	3 (2–5)	0.002

*CMR* cardiovascular magnetic resonance, *LV* left ventricle, *RV* right ventricle, *EF* ejection fraction, *EDVI* end-diastolic volume index, *ESVI* end-systolic volume index, *ED* end-diastolic, *SV* stroke volume

**Table 3:** CMR ventricular function between IPAH and SSc-PAH subgroups

Variables	IPAH (n = 20)	SScPAH (n = 17)	*p-*value
LVEF (%), median (range)	70 (46–86)	70 (54–81)	0.715
LV EDVI (ml/m^2^), median (range)	71 (44–94)	70 (54–112)	0.408
LV ESVI (ml/m^2^), median (range)	19 (7–44)	23 (10–52)	0.805
LV mass index (g/m^2^), median (range)	52 (36–72)	51 (44–68)	0.858
LV SV Index (ml/m^2^), median (range)	45 (34–58)	49 (32–71)	0.080
RVEF (%),median (range)	58 (49–79)	63 (45–73)	0.518
RV EDVI (ml/m^2^), median (range)	59 (30–102)	60 (42–111)	0.502
RV ESVI (ml/m^2^), median (range)	22 (13–49)	20 (12–61)	0.939
RV SV Index (ml/m^2^), median (range)	34 (15–60)	39 (27–54)	0.394
RV (ED) mass index (g/m^2^), median (range)	17 (9–27)	17 (14–42)	0.860
RV (ED) Mass/Volume ratio, median (range)	0.30 (0.22–0.49)	0.29 (0.23–0.41)	0.758
RV inferior wall thickness (mm), median (range)	4 (3–7)	4 (3–8)	0.262

*CMR* cardiovascular magnetic resonance, *LV* left ventricle, *RV* right ventricle, *EF* ejection fraction, *EDVI* end-diastolic volume index, *ESVI* end-systolic volume index, *ED*  end-diastolic, *SV*  stroke volume

### Myocardial oxygenation response (OS-CMR) in PAH patients

**Fig. 3:**
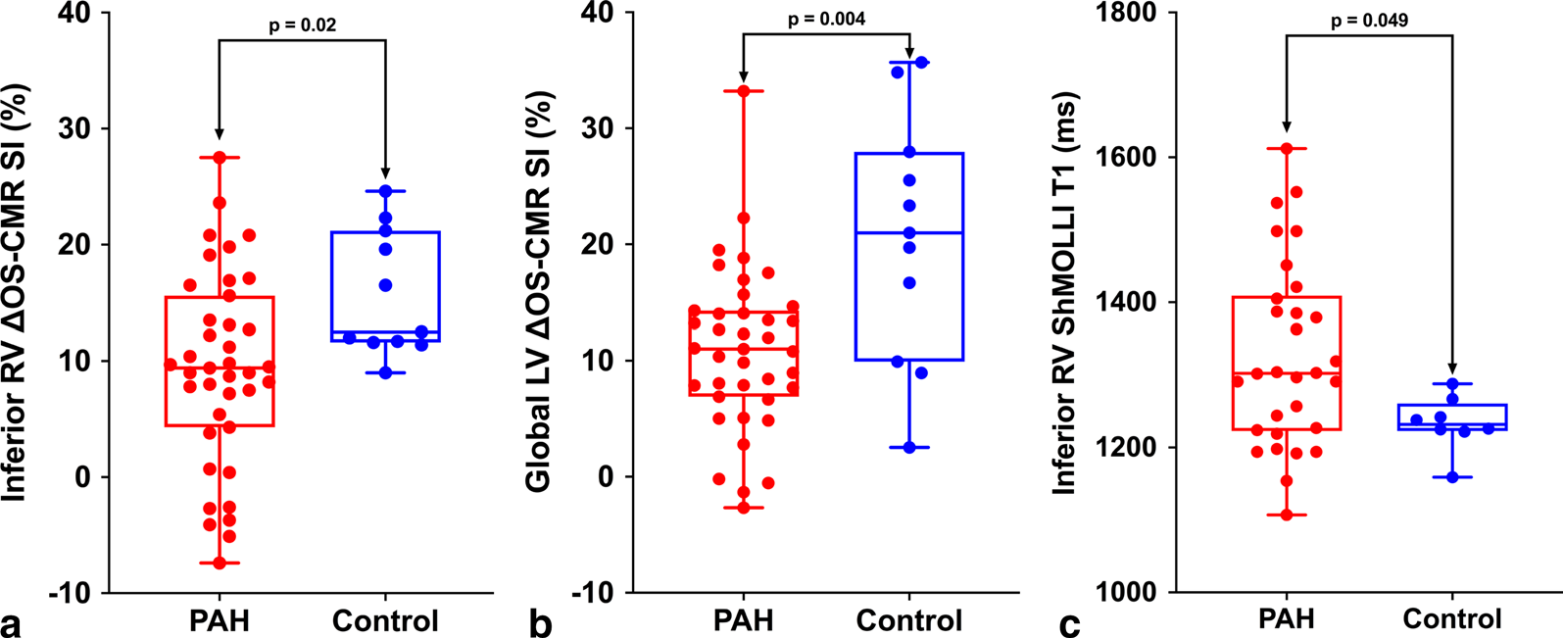
Distribution of inferior RV ΔOS-CMR signal intensity (SI), global LV ΔOS-CMR SI and inferior RV T1-mapping for PAH and healthy control group. Significant differences in inferior RV ΔOS-CMR SI (**a**), global LV ΔOS-CMR SI (**b**) and inferior RV T1-mapping values (**c**) between PAH and control

Of the total 42 PAH patients recruited, 40 (95%) patients and all the healthy controls completed the OS-CMR study protocol. The mean RV ΔOS-CMR SI change in the inferior RV segments was significantly lower in PAH group compared to the controls (9.5 (-7.4 – 42.8) vs 12.5 (9 – 24.6)%,* p* = 0.02) (Fig. 3a). The global LV ΔOS-CMR SI change was also significantly lower in PAH group compared to the controls (11.0 (-2.7 – 33.2) vs 21.0 (2.5 – 35.7)%,* p* = 0.004) (Fig. 3b). The ΔOS-CMR SI in the inferior RV wall of PAH patients was comparable to both the LV septal wall (9.5 (– 7.4–42.8) vs 11.3 (– 2.8–36.5)%, *p* = 0.32) and LV lateral wall (9.5 (– 7.4–42.8) vs 11.5 (– 9.2–31.8)%, *p* = 0.57) of PAH patients.

### Right ventricular native T1-values in PAH patients

Good quality and analysable RV native T1 maps were obtained from 71% (30/42) PAH patients and 73% (8/11) of controls. Reasons for excluding T1 maps were image artefacts and partial volume effects. Compared to healthy controls, patients with PAH had higher native T1 values in the inferior RV wall: 1303 (1107–1612) vs 1232 (1159–1288)ms, *p* = 0.049 (Fig. 3c). The LV native T1 for the healthy controls were within previously published ranges [23]. In PAH patients, the native T1 values in the inferior RV wall was comparable to both the LV septal (1303 (1107–1612) vs 1332 (1169–1492)ms, *p* = 0.71) and LV lateral wall (1303 (1107–1612) vs 1301 (1102–1500)ms, *p* = 0.85) of PAH patients. The inter-observer absolute bias was 2.0, 95% confidence interval (CI) (– 4.95, 8.95), p = 0.53 and the relative bias was – 0.05, 95% CI (– 0.19, 0.84), p = 0.41. The intra-observer absolute bias was 9.1, 95% confidence interval (CI) (– 39.12, 57.32), p = 0.68 and the relative bias was 0.48, 95% CI (– 0.28, 1.23), p = 0.18.

### Comparing RV myocardial oxygenation and RV T1 values between IPAH and SScPAH

**Fig. 4:**
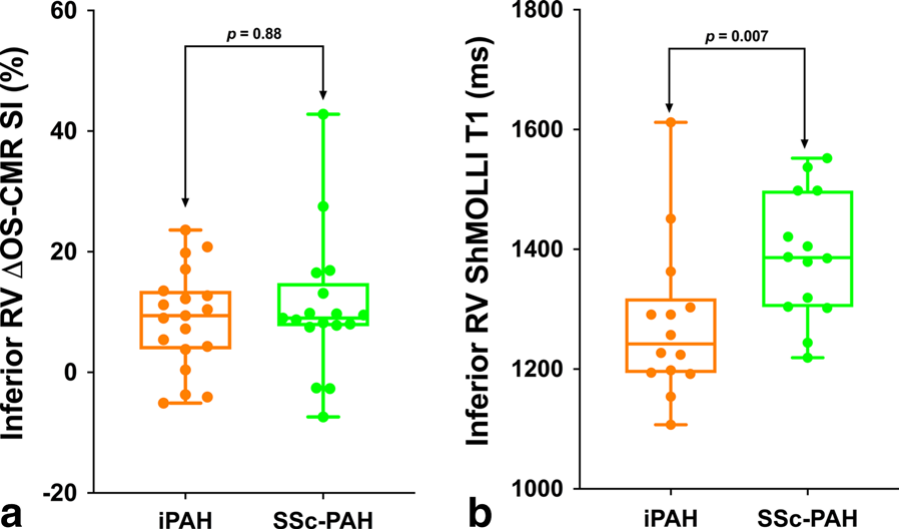
Distribution of inferior RV ΔOS-CMR SI and RV T1-mapping for IPAH and SScPAH. The inferior RV ΔOS-CMR SI (**a**) was comparable between iPAH and SSc-PAH. However there was significant difference between T1-mapping values (**b**) between iPAH and SSc-PAH

The RV ΔOS-CMR SI between the IPAH (n = 20) and SScPAH (n = 17) patients was 9.4 (– 5.1–23.6) vs 9.0 (– 7.4- 42.8)%, *p* = 0.88 (Fig. 4a). The native T1 in the inferior RV wall between the IPAH (n = 14) and SScPAH (n = 14) patients were significantly different: 1242 (1107–1612) vs 1386 (1219–1552)ms, *p* = 0.007 (Fig. 4b). Among the 5 CTEPH patients, 2/5 (40%) did not complete the OS-CMR protocol and 3/5 (60%) had poor quality RV native T1 maps, hence no comparison was made in this group.

### Correlations between RV myocardial oxygenation, RV T1 values, CMR RV volumetric/functional and RHC haemodynamic indices

**Fig. 5:**
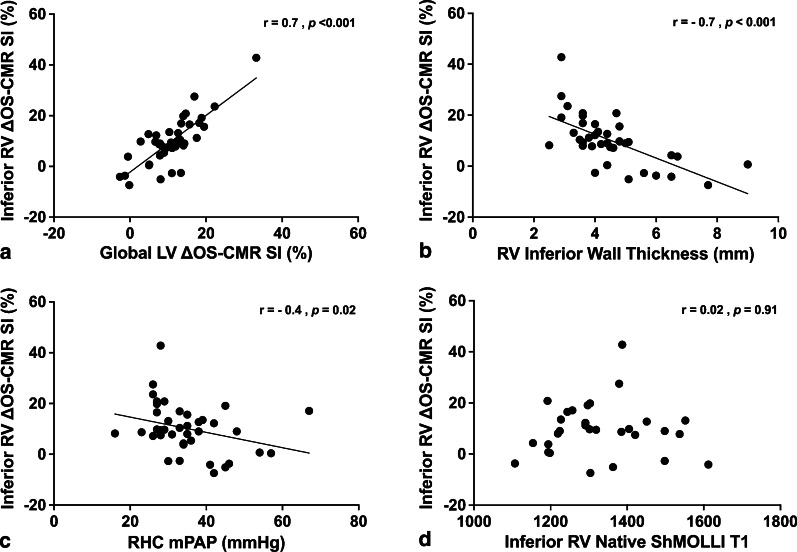
Correlation between inferior RV ΔOS-CMR SI to global LV ΔOS-CMR SI, CMR volumetric/ functional indices, hemodynamic indices and inferior RV native T1. Inferior RV ΔOS-CMR had a moderate-good correlation to global LV ΔOS-CMR SI (**a**), inferior RV wall thickness (**b**) and a moderate correlation to RHC mPAP (**c**). However no correlation demonstrated between inferior RV ΔOS-CMR and RV T1 mapping (**d**)

There was a moderate-good correlation between the inferior RV ΔOS-CMR SI and the global LV ΔOS-CMR SI (r = 0.7, *p* < 0.001) (Fig. 5a). Compared with the CMR volumetric/functional and haemodynamic indices (Additional file 1: Table S1), the inferior RV ΔOS-CMR SI had a moderate-good inverse correlation to RV inferior wall thickness (r = – 0.7, *p* < 0.001) (Fig. 5b) and moderate inverse correlation to RHC mPAP (r = – 0.4, *p* = 0.02) (Fig. 5c). This suggests that increased RV hypertrophy is associated with increased myocardial deoxygenation. However, compared to native T1 values, there was no correlation between RV ΔOS-CMR SI and RV native T1 values (r = 0.02, *p* = 0.91) (Fig. 5D). Furthermore, no correlation between RV ΔOS-CMR SI and RV native T1 values was demonstrated in IPAH and SScPAH subgroups. RV native T1 had a moderate inverse correlation to RV mass/volume ratio (r = – 0.41, p = 0.036). However, there was no other significant correlation between RV native T1 and CMR volumetric/functional or haemodynamic indices.

## Discussion

The principal finding from this study is that myocardial oxygenation response to adenosine stress is blunted in patients with PAH compared to healthy controls. The blunted oxygenation response was demonstrated in PAH patients who were stable on pulmonary vasodilator therapy and at an adaptive stage of PAH with non-obstructive epicardial coronaries, and was correlated inversely with RV wall thickness and RHC mPAP. Although we had demonstrated changes to the myocardial interstitium, no clear interaction with myocardial oxygenation was noted. These findings highlight the potential contribution of myocardial ischemia to the pathophysiology of RV dysfunction in PAH, and could subsequently lead to new potential treatment targets, in such patients.

### Right ventricular myocardial deoxygenation in PAH

In PAH, RV myocardial ischemia is acknowledged to be a significant contributor to adverse remodelling and progressive RV dysfunction [4]. Reduced right coronary artery perfusion pressure [24] and microvascular ischemia leads to progressive RV dysfunction causing RV failure and death. Abnormal pressure gradient between the aorta and the RV due to escalating pulmonary vascular resistance, reduces myocardial blood flow in the RV branch of the right coronary artery causing ischemia [7, 25]. RV myocardial ischemia is further exacerbated by inadequate compensatory increase in the myocardial vasculature owing to progressive RV hypertrophy (RVH) [7, 26, 27]. Occlusive microvascular damage and impairment in angiogenesis (known as capillary rarefaction) has been demonstrated in animal models and SScPAH, is thought to cause microvascular ischemia [5, 28]. Furthermore, a study by Meloche et al. proposes common pathobiology mechanisms between PAH pulmonary vasculature and coronary arteries [29]. These pathobiology mechanisms trigger coronary vascular remodeling in both the LV and RV leading to microvascular ischemia.

In this study, we have demonstrated a blunted myocardial oxygenation response to vasodilator stress in the inferior RV segment on PAH patients. Specifically our study demonstrates the presence in-vivo myocardial deoxygenation in the adaptive stage of PAH. The findings of in-vivo RV myocardial deoxygenation in PAH are novel because abnormalities in myocardial blood flow or microvascular dysfunction may not necessarily lead to myocardial deoxygenation [11]. Arnold et al*.* had demonstrated that in coronary artery disease, 50% of hypoperfused segments on quantitative myocardial perfusion demonstrate no evidence of deoxygenation [11]. Furthermore in dilated cardiomyopathy, Dass et al. had demonstrated disassociation between microvascular dysfunction and oxygenation [30]. Although we had not performed any direct comparison between myocardial blood flow and myocardial oxygenation, these findings provide further mechanistic insight into pathophysiology of myocardial ischemia in the RV in PAH.

The OS-CMR SI changes in the RV had good correlation with global OS-CMR SI changes seen in the LV, an area of myocardium in which there is good validation with the OS-CMR technique. In our study, the OS-CMR technique has demonstrated significant changes in the inferior RV segment. Thinned myocardium (causing partial volume effects), off-resonance artefacts and image artefacts are known limitations of the OS-CMR technique in the RV [18]. However, these limitations have previously been known to affect the accuracy of OS-CMR SI in LV myocardium [31]. Consistent voxel-size was applied during OS-CMR imaging, hence the measured inferior RV wall thickness provided sufficient coverage to avert partial volume effects. Additionally, it has been shown that the inferior RV segment has the greatest end-diastolic wall thickness once the afterload, serum biomarkers and RV structural adaptation in pre- and post-capillary pulmonary hypertension have been taken into consideration [32].

Furthermore, our study has demonstrated a moderate inverse correlation between RV OS-CMR SI and mPAP. The moderate correlation highlights that there are other pathophysiological factors (such as microvascular ischemia) that affect the RV besides elevated mPAP. Patients with microvascular ischemia often present with typical effort-induced angina, but also with atypical symptoms such as dyspnea on exertion [33]. Exertional limitation is the dominant symptom in PAH, however it is contributed by many factors such as respiratory mechanics/ventilation, cardiovascular response and psychological/emotional aspects [34]. Even though RHC resting hemodynamic measures such as mPAP and cardiac output correlate with severity of symptoms, there are considerable inter-individual variability that is not explained by RHC hemodynamic severity [35]. The findings of myocardial deoxygenation provide further mechanistic insights into the pathophysiology of myocardial ischaemia in PAH population.

### Native T1-values in the right ventricle

In PAH, the RV adaptation extends to the cardiomyocytes and interstitium altering the cardiac structure and function [8]. Although the initial adaptive changes of myocardial collagen accumulation serve as a favorable response helping the RV withstand higher pressures, over time maladaptive alteration ensues leading to fibrosis and progressive RV dysfunction [36]. Native CMR T1 parametric mapping has been used as a surrogate for diffuse interstitial fibrosis in the absence of an alternative cause of interstitial expansion (oedema, infiltrations/fibre disarray) [16]. Previous studies of RV native T1 mapping in PAH using Modified Look-Locker Inversion recovery (MOLLI) sequence has produced varying results [17, 22, 37]. One possible explanation is that each of these studies have adapted an altered version of the MOLLI sequence thus producing differing results. Utilising the ShMOLLI sequence, our study demonstrated increased native T1-values in the inferior RV wall compared to healthy controls. The native T1-values in the inferior RV wall were comparable to the LV septum and LV free wall in PAH patients, similar to the findings previously demonstrated by Spruijt et al.[22]. Unexpectedly in our study, we found that the RV native T1-values did not correlate with inferior RV OS-CMR SI changes. This is pertinent in PAH as the pressure overload leads to disruption of the healthy extracellular matrix in the RV due to the excess collagen formation and myocardial interstitial fibrosis. Changes in RV extracellular matrix could potentially disrupt the coronary microvasculature and hence myocardial oxygen supply. However, the lack of association between RV native T1-values and RV OS-CMR SI in our study suggests that the changes in RV extracellular matrix does not directly impact myocardial oxygenation.

Interestingly our study is the first to have demonstrated a significant difference in the native T1-values between the IPAH and SScPAH. We have used the ShMOLLI sequence in contrast to other studies that have used the MOLLI sequence [17, 22, 38]. The ShMOLLI sequence is heart rate independent [39] and as such is able to estimate long T1s which is important for assessing oedematous tissue [40]. Previous ShMOLLI T1 mapping studies of the LV in systemic sclerosis patients suggested the presence of interstitial fibrosis and low-grade inflammation [41]. Therefore, the higher T1-value SScPAH seen in our study could signify a combination of diffuse interstitial fibrosis and myocardial inflammation, a theory that fits with the pathophysiology of systemic sclerosis.

### Study limitations

Our study only performed a single mid-ventricular slice on OS-CMR imaging rather than the entire ventricle hence there was incomplete coverage of the full RV myocardium. This is unlikely to be of major relevance in RV assessment of PAH which is a global process. While our study had demonstrated in-vivo myocardial deoxygenation in the RV of PAH, we had not performed any direct comparison with myocardial blood flow, an area for future studies. Late-gadolinium enhancement or extracellular volume assessment was not performed due to concerns of patient safety and tolerability during CMR research scan. While OS-CMR sequence has been studied in many conditions [9], to our knowledge it has not been studied in PAH. The multiple breath-hold during rest/stress OS-CMR image acquisitions could potentially be challenging for PAH patients as dyspnoea is a common symptom in this cohort of patients. The number of subjects in the healthy control groups were relatively small especially with female controls, compared to the PAH group. However the control group were age-matched, minimizing any significant bias. This is important, especially in T1-values whereby age is known to influence native myocardial T1-values [39]. Measuring RV T1 on a curved RV myocardium can be challenging. RV T1 mapping with ShMOLLI sequence at 8 mm slice thickness can be a potential substrate for partial volume effects affecting T1-values. However, every effort was taken to ensure the ROI was drawn in the myocardium. Furthermore the adequate reproducibility and the comparable RV T1-values to the LV T1-values in PAH patients suggest accurate interpretation of RV T1. Although this study was prospective, the PAH patients were stable on pulmonary vasodilator therapy. This could have mitigated against the finding of RV ischemia. A larger study would help to determine the clinical and prognostic utility of these novel CMR techniques in PAH.

## Conclusions

Blunted OS-CMR SI suggest the presence of in-vivo microvascular dysfunction in the RV of PAH patients. Future studies are essential to determine the utility of OS-CMR of the RV in predicting progressive RV dysfunction and mortality.

## Supplementary Information

**Additional file 1: Table S1.** Correlation between right ventricular (RV) ΔOS-CMR signal intensity and RV T1-mapping to RV CMR volumetric/functional indices and right heart catheter (RHC) hemodynamic indices.

## Data Availability

The datasets generated during and/or analysed during the current study are available from the corresponding author on reasonable request.

## Abbreviations

AHA: American Heart Association
BOLD: Blood Oxygen Level Dependent
BSA: Body surface area
bSSFP: Balanced steady-state-free-precession
CI: Confidence Interval
CMR: Cardiovascular magnetic resonance
CTEPH: Chronic thromboembolic pulmonary artery hypertension
ECG: Electrocardiogram
EDVI: End-diastolic volume index
ESVI: End-systolic volume index
FOV: Field of view
IPAH: Idiopathic pulmonary artery hypertension
LV: Left ventricle/left ventricular
LVEF: Left ventricular ejection fraction
MOLLI: Modified Look-Locker Inversion recovery
mPAP: Mean pulmonary artery pressure
OS: Oxygenation-sensitive
PAH: Pulmonary arteryl hypertension
PCWP: Pulmonary capillary wedge pressure
RHC: Right heart catheterization
ROI: Region of interest
RV: Right ventricular/right ventricular
RVEF: Right ventricular ejection fraction
ShMOLLI: Shortened modified Look-Locker inversion recovery
SScPAH: Systemic sclerosis associated pulmonary artery hypertension
SI: Signal Intensity
SV: Stroke volume
TE: Echo time
TR: Repetition time

## References

[1] McLaughlin VV, McGoon MD (2006). Pulmonary arterial hypertension. Circulation.

[2] Hoeper MM, Simon R, Gibbs J (2014). The changing landscape of pulmonary arterial hypertension and implications for patient care. Eur Respir Rev..

[3] Humbert M, Sitbon O, Chaouat A, Bertocchi M, Habib G, Gressin V (2010). Survival in patients with idiopathic, familial, and anorexigen-associated pulmonary arterial hypertension in the modern management era. Circulation.

[4] Ryan JJ, Archer SL (2015). Emerging concepts in the molecular basis of pulmonary arterial hypertension: part I: metabolic plasticity and mitochondrial dynamics in the pulmonary circulation and right ventricle in pulmonary arterial hypertension. Circulation.

[5] Bogaard HJ, Natarajan R, Henderson SC, Long CS, Kraskauskas D, Smithson L (2009). Chronic pulmonary artery pressure elevation is insufficient to explain right heart failure. Circulation.

[6] Piao L, Fang Y-H, Parikh K, Ryan JJ, Toth PT, Archer SL (2013). Cardiac glutaminolysis: a maladaptive cancer metabolism pathway in the right ventricle in pulmonary hypertension. J Mol Med.

[7] van Wolferen SA, Marcus JT, Westerhof N, Spreeuwenberg MD, Marques KM, Bronzwaer JG (2008). Right coronary artery flow impairment in patients with pulmonary hypertension. Eur Heart J.

[8] Andersen S, Nielsen-Kudsk JE, Vonk Noordegraaf A, de Man FS (2019). Right Ventricular Fibrosis. Circulation.

[9] Sree Raman K, Nucifora G, Selvanayagam JB. Novel cardiovascular magnetic resonance oxygenation approaches in understanding pathophysiology of cardiac diseases. Clinical and experimental pharmacology & physiology. 2018. 10.1111/1440-1681.12916 29350784

[10] Karamitsos TD, Leccisotti L, Arnold JR, Recio-Mayoral A, Bhamra-Ariza P, Howells RK (2010). Relationship between regional myocardial oxygenation and perfusion in patients with coronary artery disease. Circulation..

[11] Arnold JR, Karamitsos TD, Bhamra-Ariza P, Francis JM, Searle N, Robson MD (2012). Myocardial oxygenation in coronary artery disease: insights from blood oxygen level–dependent magnetic resonance imaging at 3 Tesla. J Am Coll Cardiol.

[12] Grover S, Lloyd R, Perry R, Lou PW, Haan E, Yeates L, et al. Assessment of myocardial oxygenation, strain, and diastology in MYBPC3-related hypertrophic cardiomyopathy: a cardiovascular magnetic resonance and echocardiography study. European heart journal cardiovascular Imaging. 2019. 10.1093/ehjci/jey220 30668650

[13] Shah R, Parnham S, Liang Z, Perry R, Bradbrook C, Smith E (2019). Prognostic Utility of Oxygen-Sensitive Cardiac Magnetic Resonance Imaging in Diabetic and Nondiabetic Chronic Kidney Disease Patients With No Known Coronary Artery Disease. J Acad Cardiovasc Imag..

[14] Pauling L, Coryell CD (1936). The magnetic properties and structure of hemoglobin, oxyhemoglobin and carbonmonoxyhemoglobin. Proc Natl Acad Sci.

[15] Taylor AJ, Salerno M, Dharmakumar R, Jerosch-Herold M (2016). T1 Mapping: basic techniques and clinical applications. JACC..

[16] Messroghli DR, Moon JC, Ferreira VM, Grosse-Wortmann L, He T, Kellman P (2017). Clinical recommendations for cardiovascular magnetic resonance mapping of T1, T2, T2* and extracellular volume: a consensus statement by the Society for Cardiovascular Magnetic Resonance (SCMR) endorsed by the European Association for Cardiovascular Imaging (EACVI). J Cardiovasc Magn Reson.

[17] Patel RB, Li E, Benefield BC, Swat SA, Polsinelli VB, Carr JC (2020). Diffuse right ventricular fibrosis in heart failure with preserved ejection fraction and pulmonary hypertension. ESC Heart Failure.

[18] Sree Raman K, Stokes M, Walls A, Perry R, Steele PM, Burdeniuk C (2019). Feasibility of oxygen sensitive cardiac magnetic resonance of the right ventricle in pulmonary artery hypertension. Cardiovasc Diagn Ther.

[19] Piechnik SK, Ferreira VM, Dall'Armellina E, Cochlin LE, Greiser A, Neubauer S (2010). Shortened Modified Look-Locker Inversion recovery (ShMOLLI) for clinical myocardial T1-mapping at 15 and 3 T within a 9 heartbeat breathhold. J Cardiovasc Magn Reson..

[20] Cerqueira MD, Weissman NJ, Dilsizian V, Jacobs AK, Kaul S, Laskey WK (2002). Standardized myocardial segmentation and nomenclature for tomographic imaging of the heart. A statement for healthcare professionals from the Cardiac Imaging Committee of the Council on Clinical Cardiology of the American Heart Association. Circulation..

[21] Sharma P, Socolow J, Patel S, Pettigrew RI, Oshinski JN (2006). Effect of Gd-DTPA-BMA on blood and myocardial T1 at 1.5T and 3T in humans. J Magn Reson Imaging..

[22] Spruijt OA, Vissers L, Bogaard H-J, Hofman MBM, Vonk-Noordegraaf A, Marcus JT (2016). Increased native T1-values at the interventricular insertion regions in precapillary pulmonary hypertension. Int J Cardiovasc Imaging.

[23] Shah R, Sree Raman K, Walls A, Woodman RJ, Faull R, Gleadle JM (2019). Gadolinium-free cardiovascular magnetic resonance stress t1 mapping in patients with chronic kidney disease. JACC..

[24] Kahan A, Allanore Y (2006). Primary myocardial involvement in systemic sclerosis. Rheumatology.

[25] Vogel-Claussen J, Skrok J, Shehata ML, Singh S, Sibley CT, Boyce DM (2011). Right and left ventricular myocardial perfusion reserves correlate with right ventricular function and pulmonary hemodynamics in patients with pulmonary arterial hypertension. Radiology.

[26] Akasaka T, Yoshikawa J, Yoshida K, Hozumi T, Takagi T, Okura H (1996). Comparison of relation of systolic flow of the right coronary artery to pulmonary artery pressure in patients with and without pulmonary hypertension. Am J Cardiol.

[27] Murray PA, Vatner SF (1981). Reduction of maximal coronary vasodilator capacity in conscious dogs with severe right ventricular hypertrophy. Circ Res.

[28] Piao L, Fang YH, Parikh K, Ryan JJ, Toth PT, Archer SL (2013). Cardiac glutaminolysis: a maladaptive cancer metabolism pathway in the right ventricle in pulmonary hypertension. J Mol Med (Berl).

[29] Meloche J, Lampron M-C, Nadeau V, Maltais M, Potus F, Lambert C (2017). Implication of inflammation and epigenetic readers in coronary artery remodeling in patients with pulmonary arterial hypertension. Arterioscler Thromb Vasc Biol.

[30] Dass S, Holloway CJ, Cochlin LE, Rider OJ, Mahmod M, Robson M (2015). No evidence of myocardial oxygen deprivation in nonischemic heart failure. Circulation.

[31] Fischer K, Yamaji K, Luescher S, Ueki Y, Jung B, von Tengg-Kobligk H (2018). Feasibility of cardiovascular magnetic resonance to detect oxygenation deficits in patients with multi-vessel coronary artery disease triggered by breathing maneuvers. J Cardiovasc Magn Reson.

[32] Attard MI, Dawes TJW, de Marvao A, Biffi C, Shi W, Wharton J (2018). Metabolic pathways associated with right ventricular adaptation to pulmonary hypertension: 3D analysis of cardiac magnetic resonance imaging. Eur Heart J.

[33] Ong P, Camici PG, Beltrame JF, Crea F, Shimokawa H, Sechtem U (2018). International standardization of diagnostic criteria for microvascular angina. Int J Cardiol.

[34] Dumitrescu D, Sitbon O, Weatherald J, Howard LS (2017). Exertional dyspnoea in pulmonary arterial hypertension. Eur Respir Rev.

[35] Sun X-G, Hansen James E, Oudiz Ronald J, Wasserman K (2001). Exercise pathophysiology in patients with primary pulmonary hypertension. Circulation.

[36] Polyakova V, Hein S, Kostin S, Ziegelhoeffer T, Schaper J (2004). Matrix metalloproteinases and their tissue inhibitors in pressure-overloaded human myocardium during heart failure progression. J Am Coll Cardiol.

[37] Mehta BB, Auger DA, Gonzalez JA, Workman V, Chen X, Chow K (2015). Detection of elevated right ventricular extracellular volume in pulmonary hypertension using Accelerated and Navigator-Gated Look-Locker Imaging for Cardiac T1 Estimation (ANGIE) cardiovascular magnetic resonance. J Cardiovasc Magn Reson.

[38] Saunders LC, Johns CS, Stewart NJ, Oram CJE, Capener DA, Puntmann VO (2018). Diagnostic and prognostic significance of cardiovascular magnetic resonance native myocardial T1 mapping in patients with pulmonary hypertension. J Cardiovasc Magn Reson.

[39] Piechnik SK, Ferreira VM, Lewandowski AJ, Ntusi NAB, Banerjee R, Holloway C (2013). Normal variation of magnetic resonance T1 relaxation times in the human population at 1.5 T using ShMOLLI. J Cardiovasc Magn Reson.

[40] Piechnik SK, Neubauer S, Ferreira VM (2018). State-of-the-art review: stress T1 mapping—technical considerations, pitfalls and emerging clinical applications. Magn Reson Mater Phys.

[41] Ntusi NAB, Piechnik SK, Francis JM, Ferreira VM, Rai ABS, Matthews PM (2014). Subclinical myocardial inflammation and diffuse fibrosis are common in systemic sclerosis–a clinical study using myocardial T1-mapping and extracellular volume quantification. J Cardiovasc Magn Reson.

