# Supplementary material for: Right ventricular myocardial deoxygenation in patients with pulmonary artery hypertension
Source: J Cardiovasc Magn Reson. 2021 Mar 8;23:22. doi: 10.1186/s12968-020-00694-0 (PMC7938464; doi:10.1186/s12968-020-00694-0)
Supplement: Supplementary file 1 — Additional file 1: Table S1. Correlation between right ventricular (RV) ΔOS-CMR signal intensity and RV T1-mapping to RV CMR volumetric/functional indices and right heart catheter (RHC) hemodynamic indices. [file 12968_2020_694_MOESM1_ESM.docx]

**Table S1:** Correlation between right ventricular (RV) ΔOS-CMR signal intensity and RV T1-mapping to RV CMR volumetric/functional indices and right heart catheter (RHC) hemodynamic indices

| **Variable** | **Correlation** | ***p*-value** |
| --- | --- | --- |
| RV ΔOS-CMR SI | | |
| CMR RVEF | 0.2 | 0.293 |
| CMR RV EDVI | -0.1 | 0.566 |
| CMR RV ESVI | 0.17 | 0.322 |
| CMR RV mass Index | -0.2 | 0.160 |
| RV inferior wall thickness | -0.7 | <0.001 |
| RHC mPAP | -0.4 | 0.02 |
| RHC PAWP | 0.2 | 0.200 |
| RHC mRAP | 0.0 | 0.930 |
| RHC cardiac index | 0.0 | 0.978 |
| RHC PVRi | -0.1 | 0.561 |
| RV T1-mapping | | |
| CMR RVEF | 0.3 | 0.112 |
| CMR RV EDVI | 0.3 | 0.175 |
| CMR RV ESVI | <0.1 | 0.966 |
| CMR RV mass index | -0.1 | 0.535 |
| RV inferior wall thickness | -0.2 | 0.225 |
| RHC mPAP | -0.3 | 0.084 |
| RHC PAWP | 0.2 | 0.309 |
| RHC mRAP | 0.1 | 0.651 |
| RHC cardiac index | 0.3 | 0.287 |
| RHC PVRi | -0.4 | 0.114 |

CMR = cardiovascular magnetic resonance; EDVI = end-diastolic volume index; EF = ejection fraction; ESVI = end-systolic volume index; mPAP = mean pulmonary artery pressure; mRAP = mean right atrial pressure; PAWP = pulmonary artery wedge pressure; PVR = pulmonary vascular resistance; RHC = right heart catheterization; RV = right ventricular;
